# Molecular interplay of insulin resistance and cancer: advances in monoclonal antibody therapeutics

**DOI:** 10.1080/15384047.2026.2708386

**Published:** 2026-07-28

**Authors:** Sivakumar S. Moni, Fatma Ayish, Shaqraa Musawi, Ahmad Salawi, Mohamed Eltaib Elmobark, Aamena Jabeen, Mawada Abubaker Abdelgadir Mohammed, Maha Jubran Aqdi, Maram Yahya Aziabi, Raneem Hafiz Harbi, Al Anoud Mohammed Ghazwani, Sara Hassan Almalki, Raghad Abdullah Sharif, Hanin Mohammed Ezzi, Hind Mohammed Suwaydi, Taif E. Alajam, Safa Abdullah Awaji, Amwaj Yahya Marwai Nammazi, Amirah Mosa Maashi, Nesreen Ibrahim Faqiri, Atyaf Saleh Ahmed Dohal, Nahlah Salah Burayk

**Affiliations:** a Department of Pharmaceutics, College of Pharmacy, Jazan University, Jazan, Saudi Arabia; b Health Research Centre, Jazan University, Jazan, Saudi Arabia; c Internal Medicine Department, Faculty of Medicine, Jazan University, Jazan, Saudi Arabia; d Department of Medical Laboratory Technology, College of Nursing and Health Sciences, Jazan University, Jazan, Saudi Arabia; e Clinical Practice Department, College of Pharmacy, Jazan University, Jazan, Saudi Arabia; f College of Pharmacy, Jazan University, Jazan, Saudi Arabia; g Jazan University Hospital Laboratory, Jazan University, Jazan, Saudi Arabia

**Keywords:** Insulin resistance, insulin-like growth factor axis, hyperinsulinemia, tumor microenvironment, cytokine signaling, monoclonal antibody therapy

## Abstract

Insulin resistance (IR) is involved in the development, progression, and treatment resistance of cancer. Apart from contributing to obesity and type 2 diabetes, IR leads to hyperinsulinemia, disruption of insulin-like growth factor signaling, chronic inflammation, and metabolic remodeling, which fosters a pro-tumorigenic milieu. These changes stimulate the PI3K–Akt–mTOR, MAPK, JAK–STAT, and NF-κB pathways, which increase proliferation, survival, angiogenesis, immune escape, and metastasis. IR also modifies the tumor microenvironment (TME) and dampens anti-tumor immunity. IGF-1R, IL-6, IL-1β, TNF-*α*, PD-1, PD-L1, and CTLA-4 monoclonal antibodies could be beneficial by inhibiting inflammatory and oncogenic pathways and reinitiating immune surveillance. Tumor resistance and heterogeneity are significant obstacles. This is a structured narrative review of the molecular connections, antibody treatments, translational obstacles, and future refined approaches in oncology.

## 1. Introduction

Insulin resistance (IR) is a primary metabolic disorder characterized by decreased sensitivity of cells to insulin, leading to overproduction of insulin (hyperinsulinemia) and a multitude of metabolic disorders. It captures the state of impaired hormone action that elicits adaptive homeostatic defensive mechanisms that are ultimately unproductive, such as the development of excessive fat tissue, damage from fat (lipotoxicity), cell injury from oxidative stress, and chronic low-grade inflammation. These processes are interrelated and together promote cell death (apoptosis), the development of fibrous tissue (fibrosis), and a decline in the function of tissue that responds to insulin (insulin-responsive), contributing to the dysfunction of several organs. Recently, the growing importance of IR has led to recognition of its role, through mechanisms of metabolic reprogramming and inflammatory signaling, in the pathogenesis of various cancers.^1-3^ Recent and developing epidemiological and molecular studies have linked the phenomenon of IR to cancer’s incidence, progression, and mortality and are building a strong case. Further studies in large cohorts have shown that, within a twenty-year period, a patient suffering from type 2 diabetes mellitus (T2DM) has a 20-40% greater likelihood of developing cancer than that of a patient without T2DM, and that patient may develop cancer in any of the following organs: the breast, the colon, the pancreas, and the hepatocellular carcinoma. Hyperinsulinemia’s role in cancer development and growth, particularly its independent effect apart from other co-risk factors, has been shown to act both directly and indirectly, through a growth factor pathway.^4-6^ Accumulating epidemiological evidence indicates that IR and hyperinsulinemia are associated with increased risks of several malignancies, including breast, colorectal, pancreatic, hepatocellular, and endometrial cancers. However, the extent to which these associations are causative versus correlative remains an area of ongoing investigation. While substantial clinical and epidemiological evidence supports a relationship between metabolic dysfunction and cancer risk, the precise molecular mechanisms linking IR to tumor initiation and progression continue to be actively investigated.^7^ ^,^ ^8^

Insulin-like growth factors (IGFs), primarily IGF-1 and IGF-2, are members of a class of peptide hormones structurally like insulin. These hormones are crucial to growth, development, and cellular homeostasis. While IGF-1 is produced in the liver and is under the control of growth hormone, IGF-2 is produced in excess during fetal development and plays a role in tissue growth and differentiation.^9^ ^,^ ^10^ The biological activities of these ligands result from their binding to specific receptors. Most notably, the insulin-like growth factor-1 receptor (IGF-1R), which is a transmembrane receptor tyrosine kinase with a lot in common (in structure and function) with the insulin receptor (InsR). In the bloodstream, IGFs are bound to a group of proteins known as insulin-like growth factor-binding proteins (IGFBPs) that modulate the availability of IGFs, their circulating half-life, and their interaction with target tissues.^11-13^ The insulin-like growth factor (IGF) axis consists of two ligands (IGF-1 and IGF-2), their receptors (IGF-1R and IGF-2R), and a family of six high-affinity binding proteins (IGFBP-1 to IGFBP-6) that control the availability of IGFs and their activity. IGFBPs are the primary regulatory proteins of the IGF axis. All six binding proteins are expressed in human tissue and can bind to both ligands, but IGFBP-1 through IGFBP-4 are more IGF-1 selective, while IGFBP-5 and IGFBP-6 are more IGF-2 selective. IGF-1R is the main receptor involved in IGF signaling, mediating intracellular signaling through the PI3K/Akt/mTOR and MAPK/ERK pathways to promote cell proliferation and differentiation and inhibit apoptosis (anti-apoptotic effects of the PI3K pathway). IGF-2 also signals through the insulin receptor isoform A (InsR-A), which can further augment mitogenic responses. The axis, in normal physiological states, is responsible for tissue growth and metabolic homeostasis. However, alterations to this system localized to the IGF axis, such as unchanged hyper-IGFs, hypo-IGFBPs, and receptor hyperactivity, lead to unregulated proliferation and survival, enhanced angiogenesis, and tumor progression.^14-18^

A major feature of IR is dysfunction of insulin receptor substrates (InsRs), particularly IRS-1 and IRS-2. The further impairment of IRS-mediated signaling by pro-inflammatory kinases such as c-Jun *N*-terminal kinases (JNK) and IκB kinase *β* (IKKβ) diverts downstream signaling and causes metabolic dysfunction. Ironically, this dysregulation increases oncogenic signaling through compensatory activation of the IGF pathway. Research demonstrates that the overexpression of IRS-2 is linked to the higher metastatic potential of breast cancer, while the dysregulation of IRS-1 is associated with the proliferation of tumor cells.^19^ ^,^ ^20^ While IR has long been understood as a metabolic disorder, it is now considered an inflammatory disorder with chronic immune system activation. Due to the expansion of adipose tissue associated with obesity, tissue hypoxia and cellular stress develop, leading to the recruitment of immune cells (particularly macrophages and T lymphocytes). These immune cells secrete cytokines that modify insulin action in target tissues. Chronic inflammation is a characteristic feature of IR and creates an inflammatory environment conducive to tumor growth. The pro-inflammatory cytokines tumor necrosis factor alpha (TNF-*α*), interleukin-6 (IL-6), and interleukin-1 beta (IL-1β) play pivotal roles in disrupting insulin signaling and simultaneously activating oncogenic pathways.^21^ ^,^ ^22^

For example, IL-6 stimulates the Janus kinase/signal transducer and activator of transcription (JAK/STAT) pathway, leading to increased production of suppressors of cytokine signaling (SOCS) proteins. These SOCS proteins can inhibit InsR activity and enhance the proteolytic degradation of InsR proteins. Conclusively, elevated circulating IL-6, so that it is >5–10 pg/mL, counts in individuals with obesity) which is a predictive factor for the elevated incidence of cancer as well as for the worsening prognosis of the obese individuals.^23-25^ In addition, hyperinsulinemia diminishes the liver's synthesis of IGF-binding proteins (especially IGFBP-1 and IGFBP-2), thereby increasing the availability of unbound IGF-1. Higher levels of IGF-1 are significantly linked to an increased probability of developing cancer, with meta-analyzes showing a 1.3- to 1.5-fold increased likelihood of breast and prostate cancer in those with high levels of circulating IGF-1. Enhanced IGF signaling, in turn, promotes cell cycle progression, inhibits cell death (apoptosis), and promotes new blood vessel formation (angiogenesis) via cross-talk with vascular endothelial growth factor (VEGF) pathways.^26^

Dynamic interactions among cancer and immune system cells in the tumor microenvironment (TME) are pivotal to understanding how the TME disrupts IR and the insulin-like growth factor (IR-IGF) axis. Tumor-associated macrophages (TAMs), especially the M2 class, are reported to produce cytokines and growth factors that amplify IGF activity but weaken anti-tumor activity. Concomitantly, cancer cells undergo metabolic reprogramming that, in the context of IR, increases glucose uptake and aerobic glycolysis, thereby driving tumor progression.^27^ ^,^ ^28^

Inflamed TME also induces changes in the types of immune cells present, while maintaining strong M1 macrophage and effector T cell responses. In tandem with failed adipose tissue, these cells secrete pro-inflammatory cytokines that cause IR and promote apoptosis of immune cells by activating stress and inflammatory signaling pathways. In the TME, further impairment of IRS signaling blunts the PI3K/Akt pathway, which, in a persistent inflammatory state, is sustained, thereby shifting the TME toward a pro-tumorigenic state by promoting cellular growth, the formation of a new blood supply, supporting tumor progression and survival, and conferring resistance to apoptosis. IR and cancer progression operate through overlapping mechanisms that require a systems approach.^29-31^ The role of IR and related signaling pathways in cancer progression is supported by extensive mechanistic and preclinical research. However, the strength of this evidence varies across molecular targets. For some of these targets, evidence is based on direct experimental validation. For others, evidence is more indirect, based on translational studies or indirect clinical associations. Thus, the distinction between established clinical evidence and preclinical research or emerging therapeutic hypotheses is essential for analyzing therapeutic options that address the IR–cancer association.^29-31^

The central role of the IR–IGF axis in cancer biology has emerged as a promising therapeutic target. Monoclonal antibodies (mAbs) are highly specific, laboratory-engineered immunoglobulins designed to recognize and bind to a single antigenic epitope, thereby enabling precise targeting of disease-associated molecules. In the context of IGF signaling, mAbs directed against IGF-1R, such as figitumumab and ganitumab, have been developed to inhibit receptor activation and downstream signaling. Clinical trials have demonstrated varying degrees of efficacy, with response rates ranging from 10% to 30% in selected patient populations, highlighting both the potential and challenges of targeting this pathway. Monoclonal antibodies directed against several targets, including cytokines and chemokines, vascular endothelial growth factor (VEGF), PD-1, PD-L1, CTLA-4, and other immune checkpoint molecules, have attracted interest as potential cancer therapies. This collection of medications may alter cancer-related inflammation and immune metabolism and address immunologic and vascular pathways of cancer. While this collection of therapies has shown some promise, the diversity among patient populations and the relative lack of biologically informative predictive markers have limited their success in clinical practice. In addition to ligand–receptor blockade, these antibodies facilitate receptor internalization and degradation, thereby suppressing sustained oncogenic signaling. Furthermore, emerging strategies involve combination therapies integrating IGF-1R-targeting mAbs with EGFR inhibitors, PI3K/Akt pathway modulators, or immune checkpoint inhibitors, aiming to overcome resistance and improve therapeutic outcomes. Resistance mechanisms, including compensatory activation of InsR-A and cross-talk with other receptor tyrosine kinases (e.g., EGFR), underscore the need for such rational combination approaches.^30-32^ In addition to directly inhibiting IGF signaling, new approaches focus on modifying the insulin-resistant TME by targeting inflammatory mediators, metabolic control, and immune checkpoint pathways. Combining monoclonal antibody treatments with metabolic approaches may help increase efficacy and reduce off-target effects. Several issues still need to be addressed, including the need for actionable predictive biomarkers, more precise patient stratification, and the management of treatment resistance. Most studies that investigate IR, IGF signaling, and cancer progression do so without considering the relationships and therapeutic mechanisms among these areas.

This article critically examines the role of IR in cancer progression, highlighting the complex interplay among IGF signaling, inflammatory cytokines, and the TME in driving oncogenic processes. It places particular emphasis on key signaling pathways, including IRS–PI3K/Akt/mTOR, MAPK, JAK–STAT, and NF-κB, as well as emerging molecular targets with therapeutic potential. The discussion distinguishes established clinical evidence from preclinical findings and emerging therapeutic hypotheses, providing a balanced and evidence-based perspective. Special attention is given to the development, therapeutic efficacy, limitations, and resistance mechanisms of monoclonal antibody-based strategies targeting the IR–tumor axis, particularly those directed against IGF-1R, inflammatory cytokines, angiogenic mediators, and immune checkpoint pathways.^33^ ^,^ ^34^ Furthermore, translational challenges, biomarker-guided patient stratification, and future opportunities for combination therapeutic approaches integrating targeted, metabolic, and immunological interventions are explored. Collectively, these insights provide a clinically relevant framework for advancing precision oncology strategies to overcome IR-associated cancer progression.

## 2. Methodology

### 2.1. Literature search strategy

A comprehensive literature review was conducted to examine the molecular crosstalk of IR, the insulin-like growth factor (IGF) axis, and cancer progression. The primary literature review included the following databases: PubMed, Scopus, Web of Science, and Google Scholar, as well as studies conducted between January 2000 and March 2026, to capture the breadth of foundational studies and more recent developments in immunometabolism and oncological signaling research. The search encompassed both controlled and uncontrolled vocabulary, with a specific focus on IR, IGF signaling, cytokine signaling, and immunotherapeutic targeting. The search strategy focused on several key phrases, including IR, Insulin-like growth factor, Cytokine-mediated signaling, immune–metabolic crosstalk, IRS phosphorylation, PI3K/Akt/mTOR, JAK/STAT, NF-κB pathways, TME, mAbs, and cancer IR-targeted therapy.

### 2.2. Study selection and eligibility criteria

The studies were selected systematically to demonstrate transparency and high-quality research. After duplicates were removed, the titles and abstracts were screened using defined criteria focusing on IR, IGF axis signaling, cytokine interactions, and therapeutic targeting in cancer. Their eligibility was evaluated based on their full-text articles. Studies were included if they focused on the molecular mechanisms of the IR-IGF axis, immune-metabolic crosstalk, and therapeutic interventions, especially the mAbs. Studies were excluded if they lacked a mechanistic focus, were non-peer-reviewed, or had incomplete data. Using a structured methodology, data were extracted based on study design, key molecular targets (such as IGF-1R, IRS proteins, cytokines), the signaling pathways involved (such as PI3K/Akt/mTOR, MAPK, JAK/STAT, NF-κB), therapeutic agents, and quantitative variables. The data were synthesized in an evaluative way to find the major therapeutic and mechanistic patterns.

### 2.3. Evidence assessment

As this article was conducted as a structured narrative review rather than a systematic review or meta-analysis, a formal risk-of-bias assessment was not performed. Included studies were evaluated according to their scientific relevance, methodological rigor, study design, clinical applicability, publication date, and contribution to understanding insulin resistance, IGF-axis dysregulation, inflammatory mediators (including IL-1β, IL-6, and TNF-*α*), tumor microenvironment remodeling, and monoclonal antibody therapeutics. Evidence was interpreted according to its level of support, including clinical evidence, preclinical findings, indirect associations, and emerging therapeutic hypotheses.

## 3. Insulin resistance, tumor microenvironment, and metabolic reprogramming

The TME is a complex, dynamic, and diverse ecosystem comprising cancer cells, stroma, immune constituents, extracellular matrices, and soluble signaling factors. It is becoming clear that IR and associated chronic inflammatory states significantly impact the TME. These changes assist in the initial formation of tumors, their progression, and resistance to tumor therapies. TME should not be considered a passive component of the tumor. Instead, the TME reprograms cellular constituents to promote tumor growth.^35^ ^,^ ^36^

### 3.1. Clinical relevance of insulin resistance in cancer

The clinical significance of IR continues to broaden, with growing recognition of IR as an important metabolic disorder that encompasses all forms of cancer evolution and progression, as well as disturbances in glucose metabolism. Other conditions related to IR that occur in clinical practice, such as obesity, visceral obesity, hyperinsulinemia, and metabolic syndrome, are also associated with an increased incidence of breast, colorectal, pancreatic, hepatocellular, endometrial, and prostate cancers. In IR, persistent hyperinsulinemia increases insulin and IGF-1 concentrations. This promotes binding to the InsR-A and the IGF-1R, thereby activating them. This leads to activation of the mTOR and PI3K/Akt pathways and the MAPK signaling pathways, which then promote cellular proliferation, angiogenesis, and cellular metabolic resistance to apoptosis. This constitutes an adequate condition for the initiation and progression of cancer.^17-23^ ^,^ ^29-33^

IR, the most prevalent form of metabolic dysfunction, is a critical factor in the incidence and evolution of cancers. In clinical and epidemiological literature, one of the most common proxy indicators of insulin resistance is found in the Homeostatic Model Assessment of Insulin Resistance (HOMA-IR). Derived from the levels of fasting plasma glucose (FPG) and fasting insulin (FI), HOMA-IR provides an indirect estimate of IR. Higher HOMA-IR values exhibit a positive association with the incidence of breast, colorectal, pancreatic, endometrial, and hepatocellular cancers. The persistent presence of IR leads to hyperinsulinemia and the aggressive stimulation of InsR and IGF-1R signaling. Consequently, the activated signaling pathways of InsR and IGF-1R trigger the PI3K/Akt/mTOR and MAPK pathways, which ultimately propel cellular proliferation, angiogenesis, and metabolic resistance to apoptosis. Thus, HOMA-IR may provide an estimate of tumor-related metabolic dysfunction and resistance to apoptosis and may serve as a surrogate biomarker for identifying patients with activation of the IR–IGF axis who could benefit from therapies targeting this signaling network.^1-3^ Chronic hyperinsulinemia and HOMA-IR elevate InsR-A and IGF-1R and their downstream pathways of cancer. There is an argument that HOMA-IR can serve as a marker of metabolic dysfunction and a predictive tool for patients with IR–IGF axis dysfunction, and therefore for those most likely to respond to therapy, including proposed therapies that utilize monoclonal antibodies against IGF-1R and other pathways in this signaling cascade.^17-23^ ^,^ ^29-33^

Glycated hemoglobin (HbA1c) reflects the average blood glucose concentration over the last three months and is a widely accepted clinical biomarker for long-term glucose control. HbA1c is not a direct measure of IR, but high HbA1c is suggestive of chronic hyperglycemia and metabolic dysfunction. Emerging studies have identified a positive association of elevated HbA1c levels with cancer incidence, cancer mortality, and poor treatment response. Chronic hyperglycemia can cause oxidative stress, DNA damage, and sustained production of inflammatory cytokines, which can lead to a metabolic switch in tumor and stromal cells. These changes help activate NF-κB, JAK/STAT, and PI3K/Akt/mTOR signaling pathways, which in turn promote tumor cell expansion and support tumor immune evasion. As a result, HbA1c could potentially be incorporated into precision oncology frameworks that aim to combine immunotherapy and metabolic targeting of cancer.^7-9^ Obesity, especially visceral adiposity, is a strong clinical indicator of insulin resistance as well as the development of cancer. Subcutaneous adipose tissue and visceral fat are not the same. Unlike subcutaneous fat, visceral fat is an endocrine and immunological organ. Visceral adipose tissue functions as an active endocrine and immunological organ that secretes adipokines, inflammatory cytokines, chemokines, and growth factors. Macrophage recruitment, immunosuppression in the tumor microenvironment, activation of tumor-associated macrophages, and extracellular matrix remodeling are all effects of chronic inflammation in visceral fat. There is a strong association between breast, colorectal, pancreatic, hepatocellular, endometrial, and renal cancers and visceral obesity. The roles of IR and insulin/IGF signaling vary across cancers. Each tumor type has distinct metabolic dependence, microenvironment, immune composition, and response to targeted therapies.^21^ ^,^ ^23^ ^,^ ^25^ ^,^ ^30-32^ Furthermore, inflammation induced by obesity can alter immune cell function, cytokine levels, and monoclonal checkpoint therapy, and therefore may also affect the response to monoclonal antibody therapy. Visceral fat may then be measured using waist circumference or various imaging techniques. These evaluations may reflect cancer risk and the likelihood of responding to cancer therapies.^10-12^

Metabolic dysfunction-associated steatotic liver disease (MASLD) provides a clinically valuable framework for analyzing the relationship between IR and cancer. Sustained IR causes hepatic steatosis, lipotoxicity, oxidative stress, and chronic inflammation, culminating in progressive fibrosis and collectively establishing a pro-carcinogenic hepatic microenvironment. These persistent changes, both metabolic and inflammatory, ultimately influence the onset of hepatocellular carcinoma by the coordinated stimulation of insulin/IGF and inflammatory cytokines and the activation of neoplastic pathways, such as the PI3K–Akt–mTOR and NF-κB pathways. This further indicates the need to combine metabolic therapeutic strategies with standard approaches to cancer treatment to benefit patients.^37^ Although epidemiological evidence consistently supports the association between IR and increased cancer risk, the predictive value of clinical metabolic biomarkers for guiding monoclonal antibody therapy remains under investigation and requires prospective clinical validation.

### 3.2. Insulin resistance as a driver of tumor-promoting microenvironment

**Figure 1.**
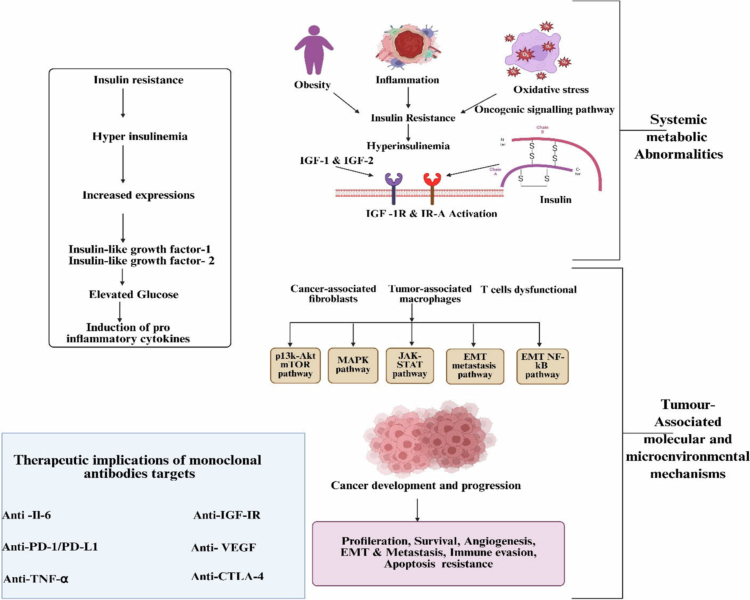
Systemic metabolic abnormalities and tumor-associated molecular mechanisms linking insulin resistance to cancer progression and therapeutic targets. Systemic metabolic abnormalities, including obesity, chronic inflammation, oxidative stress, and insulin resistance, promotde hyperinsulinemia, elevated glucose levels, increased insulin-like growth factors (IGF-1 and IGF-2), and the production of pro-inflammatory cytokines. Hyperinsulinemia activates the insulin receptor isoform A (InsR-A) and the insulin-like growth factor-1 receptor (IGF-1R) in tumor cells, triggering oncogenic signaling pathways, including PI3K–Akt–mTOR, MAPK, JAK–STAT, NF-κB, and epithelial–mesenchymal transition (EMT)-associated signaling. Concurrently, insulin resistance remodels the tumor microenvironment (TME) through interactions with cancer-associated fibroblasts (CAFs), tumor-associated macrophages (TAMs), dysfunctional T cells, and inflammatory mediators, thereby promoting tumor proliferation, survival, angiogenesis, EMT, metastasis, immune evasion, and resistance to apoptosis. The figure also highlights major monoclonal antibody therapeutic targets, including IGF-1R, IL-6, TNF-*α*, VEGF, PD-1/PD-L1, and CTLA-4, which represent potential strategies for biomarker-guided precision cancer therapy in insulin resistance-associated malignancies.

In IR states, persistent hyperinsulinemia and elevated circulating IGFs drive proliferative signaling in the TME and tumor-associated stroma (TAS). Continuous activation of the isoform A of the InsR-A and the IGF-1R elicits the PI3K/Akt/mTOR and the MAPK signaling cascade pathways that facilitate the proliferation and survival of the cells, as well as the metabolism and angiogenesis of the cells. In the TME, IL-6, TNF-*α*, IL-1β, and other cytokines amplify oncogenic pathways and create a web of signaling that drives tumor progression, represses cellular metabolism, and promotes resistance to therapy.^38-40^ In chronic low-grade inflammation, Hyperinsulinemia, and IR, persistent inflammation triggers sustained production of inflammatory cytokines such as TNF-*α*, IL-6, and IL-1β, which disrupt insulin signaling and promote tumor promotion, angiogenesis, epithelial-mesenchymal transition (EMT), and immune evasion. IR, therefore, establishes a chronic low-grade inflammatory state with TNF-*α* as a background, creating both systemic and localized environments that promote malignant transformation.^41^ ^,^ ^42^ The molecular pathways linking insulin resistance to tumor development and progression are summarized in Figure 1.

### 3.3. Remodeling of immune cells within TME

A major characteristic of the TME is the presence of numerous immune cells that support tumor proliferation and dampen anti-tumor immune activities. In the context of IR, immune cell composition undergoes significant modification. This is evident from the increased infiltration of TAMs, MDSCs, and Tregs, coupled with decreased activity of cytotoxic T lymphocytes. Macrophages, especially the M2 phenotype, are instrumental at the intersection of metabolic dysregulation and cancer. They produce growth factors, cytokines, and matrix remodeling enzymes, such as IL-6 and IL-10, that stimulate tumor growth and create an immunosuppressive microenvironment.^43^ ^,^ ^44^

The remodeling of the immune TME is largely driven by the persistent pro-inflammatory triggers associated with IR. Regarding IR, there is increased production of TNF-*α*, IL-6, and IL-1β. These cytokines stimulate interrelated signaling pathways, including JAK-STAT, NF-κB, p38 MAPK, and JNK. Importantly, these pathways do not function independently; they create an integrated signaling system that promotes immune evasion, metabolic reprogramming, and tumor growth.^45^ ^,^ ^46^ Among these, signal transduction involving IL-6 and its receptor complex (IL-6Rα/gp130) is the primary pathway for activating JAK1/2, which leads to phosphorylation of the oncogenic transcription factor STAT3. Phosphorylated STAT3 is transported to the nucleus, where it initiates transcription of genes that promote tumor development. It transcriptionally activates the anti-apoptotic proteins Bcl-2 and Bcl-xL, which prolong cell survival, and activates cyclin D1, which accelerates the cell cycle and increases cell proliferation. It also increases the rate of angiogenesis by regulating VEGF, thereby supplying nutrients and oxygen to growing tumors. These collective effects of STAT3 place it in a major role in modulating tumor growth, survival, and vascularization within the TME.^47^

In the context of immune resistance, JAK–STAT signaling via IL-6 provides transcriptional instruction sets that promote processes in tumors, thereby bolstering the growth of resistant malignant cell populations. Furthermore, STAT3 activation is significant in metabolic reprogramming in tumor cells. It promotes the expression of enzymes that carry out glycolysis and cellular respiration, thereby providing the tumor with the metabolic flexibility needed to increase cellular proliferation. Additionally, the STAT3 signaling pathway promotes the proliferation of immunosuppressive cell types, such as Tregs and MDSCs, thereby further aiding immune-evasion mechanisms within the TME. These studies highlight the IL-6-JAK-STAT pathway, which underlies tumor metabolic dysregulation and promotes tumor progression.^48-50^

The activation of a multiprotein complex known as the NLRP3 inflammasome, located in the cytosol, triggers the production of IL-1β. The NLRP3 inflammasome is responsible for detecting and responding to cellular stress caused by the accumulation of certain lipids, mitochondrial dysfunction, and oxidative stress. Once activated, NLRP3 inflammasome complexes are adjacent to a pro-IL-1β molecule and trigger its activation via caspase-1.^51^ ^,^ ^52^ An active form of IL-1β is secreted and can induce a response by binding to the IL-1 receptor (IL-1R1). This, in turn, leads to the recruitment of MyD88, which initiates a sequence of downstream events that culminate in the activation of the NF-kappa B (NFB) and Mitogen-Activated Protein (MAP) kinase (MAPK) pathways. These NFB and MAPK pathways lead to the release of additional proteins/antibodies (the products of the genes), which contribute to and sustain inflammation and exacerbate the activation of stress-associated proteins (antibodies) such as JNK and p38 MAPK. It is recognized that IL-1β signaling by itself does not cause the stimulation of JAK-STAT pathways. However, signaling can stimulate and increase JAK-STAT pathways by elevating numerous other cytokines, most notably IL-6. JAK and STAT pathways are not activated by IL-1β; therefore, inflammasome activation increases the production of numerous cytokines that contribute to the disruption of insulin signaling.^53-55^ Table 1 illustrates the components of the cytokine-immune-metabolic signaling network in the TME affected by IR, including prominent immune cell types and cytokines, as well as the relevant intracellular signaling pathways, all of which facilitate immune evasion, metabolic reprogramming, and tumor progression. Additionally, the table underscores the convergence of JAK-STAT, NF-κB, and stress kinase pathways as primary controllers that integrate inflammation and cancer-promoting metabolic reprogramming.

**Table 1.** IR signaling network remodeling the TME and cancer progression.

Cellular/Molecular entity	Description	References
TAMs, M2	The M2 macrophage-associated TAM subtype plays an important role in immunometabolism regulation in the TME, as it is primarily derived from adipose tissue-associated and infiltrating macrophages. These macrophages secrete the pro-tumorigenic cytokines TNF-*α*, IL-6, and IL-10, which, in turn, activate major signaling pathways like JAK–STAT3, NF-κB, and JNK. The activation of these pathways modulates downstream transcription factors, such as STAT3 and NF-κB, leading to increased rates of glycolysis and lipid remodeling. These changes create an immunosuppressive microenvironment that promotes tumor growth and progression and facilitates immune evasion.	[44,45]
Myeloid-Derived Suppressor Cells (MDSCs)	MDSCs originate from bone marrow and play an important role in immunosuppression in the TME. MDSCs are involved in the production of IL-6 and TGF-*β*, which lead to the activation of JAK-STAT3 and PI3K-Akt. These MDSCs, in collaboration with mTOR and STAT3, induce metabolic changes that promote tumor growth. Functionally, they inhibit T-cell activity, confer immune escape, and consequently sustain tumor progression.	[44,45]
Regulatory T Cells (Tregs)	Tregs are a specialized group of adaptive immune cells that help the immune system tolerate the presence of tumors. Tregs promote tumor growth by releasing the immunosuppressive cytokines IL-10 and TGF-*β*, which activate the STAT3 and SMAD pathways. Through the crucial transcription factors FOXP3 and STAT3, Tregs are thought to modulate immune metabolism and inhibit effector T cell activity. This Treg activity reduces tumor immunity and increases tolerance to tumors, thereby aiding tumor survival.	[44,45]
TNF-*α* Axis	The TNF-*α* axis, especially when stimulated by macrophages and adipocytes, is pivotal in linking inflammation to IR and tumor progression. TNF-*α* initiates several key signaling pathways, including JNK, NF-κB, and MAPK, which then alter the behavior of key molecular players such as IRS-1 and AP-1. These changes to the signaling pathways weaken InsR signaling and activate specific inflammatory centers. Functionally, TNF-*α* drives epithelial–mesenchymal transition (EMT), exacerbates tumor invasion and chronic inflammation, and ultimately promotes cancer progression in the TME.	[46,47]
IL-6 Axis	The IL-6 axis, primarily derived from TAMs and stromal cells, is a major contributor to tumor progression under insulin-resistant conditions. IL-6 activates the JAK1/2–STAT3 pathway, which affects upstream and downstream targets, including STAT3 and SOCS3. This pathway is associated with metabolic reprogramming, including increased glycolysis and mitochondrial changes. From a functional perspective, the IL-6–STAT3 axis drives tumor cell proliferation and angiogenesis, contributing to tumor growth and microenvironmental survival via VEGF.	[47-51]
IL-1β/Inflammasome Axis	In the TME, the NLRP3 inflammasome links metabolic stress to inflammatory signaling via the IL-1β/inflammasome axis. When the NLRP3 inflammasome is activated, IL-1β is matured by caspase-1, and, via MyD88, IL-1β activates NF-κB and the stress kinases p38 MAPK and JNK. This is coupled with the induction of oxidative stress and metabolic dysfunction, which drive further inflammation. This axis also facilitates angiogenesis, tumor invasiveness, and perpetuation of the cytokine storm, thereby driving tumor development.	[52-55]
Cancer Cells	Cancer cells within the TME exhibit intrinsic metabolic and signaling adaptations that support tumor progression. These tumor cells produce autocrine factors such as IL-6 and VEGF, thereby activating key pathways, including STAT3, PI3K–Akt–mTOR, and HIF-1α. This signaling regulates molecular targets such as GLUT1, HK2, and VEGF, driving enhanced glucose uptake and aerobic glycolysis (Warburg effect). Collectively, these metabolic and signaling alterations promote tumor cell proliferation, survival, and metastasis.	[40-42,47-51]
Stromal Cells (TAS)	Tumor-associated stroma (TAS), such as fibroblasts and endothelial cells, plays supportive roles in tumor development. They produce cytokines like IL-6 and VEGF, which activate the STAT3 and MAPK pathways. Angiogenesis and nutrient supply are stromal cell functions mediated by the regulation of ECM proteins and VEGF. Overall, these processes help the tumor expand and provide structural support within the TME.	[40,41,45]
Cytokine Crosstalk Network	The network of crosstalk among cytokines in the tumor microenvironment is a multi-cell signaling and communication system driven by inflammatory agents such as TNF-*α*, IL-6, and IL-1β. These cytokines initiate a multitude of intertwined pathways, including the JAK-STAT, NF-κB, and JNK pathways. These pathways are responsible for the integrated control and modulation of transcription factor expression, such as STAT3 and NF-κB. This perturbed, integrated signaling network alters cellular metabolism and maintains a state of ongoing inflammation, ultimately driving tumor development and enabling the tumor to evade the immune system.	[46-51]

### 3.4. Metabolic reprogramming in the TME

The hallmark of cancer is metabolic reprogramming, which is a significant consequence of cytokine signaling in the insulin-resistant TME. In IR, the combination of chronic inflammation, sustained activation of pathways such as JAK-STAT, NF-κB, and JNK, and reprogramming of cellular metabolism enables tumor cells to sustain rapid proliferation and resist apoptosis, even under adverse microenvironmental conditions. The metabolic changes described above are not independent but are closely linked to the immune dysregulation and cytokine signaling changes discussed earlier.^56-58^ The prominent feature of metabolic re-programming in cancer is the shift towards aerobic glycolysis, known as the Warburg effect. Tumor cells primarily convert glucose to lactate, even when there is ample oxygen to perform oxidative phosphorylation. This shift in metabolism is due to the activation of transcription factors induced by cytokine signaling, such as STAT3 and HIF-1α, which increase the expression of key glycolytic enzymes like hexokinase-2 (HK2) and glucose transporter-1 (GLUT1). In IR, elevated IL-6 further activates STAT3, increasing glycolytic flux and promoting metabolic flexibility. This reprogramming supports rapid ATP production and provides intermediates to biosynthetic pathways needed to sustain tumor growth.^59-61^

Beyond glycolysis, lipid processing changes considerably within the insulin-resistant TME. Hyperinsulinemia, along with the persistent inflammatory response, increases lipogenesis and fatty acid synthesis by activating the PI3K–Akt–mTOR pathway. Increased use of these pathways by tumor cells supports the synthesis of both membrane and signaling lipids essential for their proliferation and survival. Furthermore, fatty acids released from adipocytes in the TME provide an alternative energy source that increases metabolic flexibility TAMs and stromal cells contribute to this process by releasing metabolites and cytokines, including TNF-*α* and IL-6, that drive lipid remodeling and support metabolic reprogramming.^62^ ^,^ ^63^ Mitochondrial function is also regulated dynamically in cancer cells under insulin-resistant conditions. Although glycolysis is regulated, mitochondrial function is not entirely suppressed. Rather, mitochondrial function is reprogrammed to facilitate anabolic processes and to maintain the redox balance. Cytokines of the IL-1β family, particularly those that activate the NF-κB pathway, promote mitochondrial stress and reactive ROS generation. These ROS serve as second messengers, activating MAPK, STAT3, and other oncogenic pathways, thereby establishing a positive feedback mechanism that maintains integrated metabolic and inflammatory signaling. The combination of mitochondrial dysfunction and cytokine signaling is a major component of the immunometabolic landscape of cancer.^64-66^

Metabolic reprogramming is also driven by competition for nutrients in the TME between tumor and immune cells. Due to their high glucose uptake and consumption, cancer cells deprive effector immune cells, especially cytotoxic T lymphocytes, of critical metabolic building blocks. Such a process is referred to as metabolic competition, and it exacerbates T-cell exhaustion and dysfunction of anti-tumor immunity. At the same time, MDSCs and Tregs, which are immunosuppressive cells, are modified to survive the metabolic stress of low-glucose, high-lactate conditions, thereby altering their immune-escape properties. Thus, metabolic reprogramming not only fuels tumor growth but also undermines immune surveillance.^67-69^ Rapidly growing tumors exhibit hypoxia, which worsens metabolic adaptation. Anaerobic conditions stabilize HIF-1α, which regulates the transcription of genes involved in glycolysis, blood vessel formation, and cell viability. In cases of IR, cytokines such as IL-6 and TNF-*α* collaborate with hypoxic signaling to increase HIF-1α levels, thereby driving angiogenesis via VEGF. This promotes nutrient supply and accelerates tumor expansion. The fusion of hypoxic and inflammatory signaling highlights the complexity of the metabolic changes in the TME.^70-72^

Cancer cells can induce modifications in the surrounding microenvironment, including changes in the metabolism of both cancer and stromal cells, that lead to further disease progression and the acquisition of resistance mechanisms to therapy. These metabolic alterations enhance cell survival and reduce the efficacy of therapeutic approaches by meeting the cells' bioenergetic and biosynthetic requirements.^57^ ^,^ ^58^ Notably, reactivation of the PI3K–Akt–mTOR and STAT3 signaling pathways has been associated with resistance to therapeutic mAbs targeting the IGF axis, further emphasizing the importance of metabolic signaling in the development of therapeutic resistance. Several factors, such as intratumoral hypoxia, extracellular matrix (ECM) remodeling, and cellular interactions between immune and cancer cells, alter insulin signaling and contribute to metabolic reprogramming in the insulin-resistant TME.^45^ ^,^ ^69-72^ Convergence of the JAK–STAT, NF-κB, and PI3K–Akt–mTOR signaling pathways strengthens an interconnected signaling network that fuels tumor growth, immune evasion, and resistance to therapy.^49-51^ Consequently, the development of novel therapeutic approaches targeting synergistic immunometabolic pathways offers a unique opportunity to improve treatment efficacy by reprogramming tumor metabolism and modulating the underlying inflammatory signaling.

## 4. Monoclonal antibody therapeutics targeting the insulin resistance–tumor axis

The combined effects of inflammatory responses, chronic inflammation, and alterations in the TME create several potential molecular targets for therapeutic intervention. mAbs are especially valuable due to their high specificity, target selectivity, and ability to disrupt extracellular ligand–receptor interactions central to oncogenic signaling. Within the IR–tumor axis, mAbs can simultaneously target the IGF pathway, cytokine signaling networks, and immune checkpoints, which is highly relevant given the multidimensional complexity of tumor progression.^73^ ^,^ ^74^

IR is characterized by persistent hyperinsulinemia, dysregulated IGF signaling, and sustained inflammatory cytokine production, thereby maintaining oncogenic driver pathways including PI3K–Akt–mTOR, JAK–STAT, and NF-κB. Because many of these pathways are initiated through extracellular ligands and receptors, they are amenable to antibody-based inhibition. Unlike small-molecule inhibitors, mAbs generally exhibit greater target specificity and fewer off-target effects, particularly when directed against receptor tyrosine kinases or soluble cytokines. In addition, mAbs may induce receptor internalization, ligand neutralization, and antibody-dependent cellular cytotoxicity (ADCC), resulting in both direct anti-tumor and immune-modulatory effects. This dual activity is especially beneficial in insulin-resistant tumors, where both metabolic signaling and immune evasion contribute to disease progression.^75-79^

The IGF axis plays a significant role in IR-induced tumorigenesis. Overactivation of IGF-1R signaling promotes tumor proliferation, survival, and resistance to apoptosis through the PI3K–Akt–mTOR and MAPK pathways. Monoclonal antibodies such as figitumumab and ganitumab have been developed to target IGF-1R by inhibiting ligand binding and receptor activation. These antibodies suppress IGF-mediated signaling by inducing receptor downregulation and subsequent degradation. However, clinical outcomes have been inconsistent, primarily due to compensatory stimulation of InsR-A and crosstalk with other receptor tyrosine kinases such as EGFR. These findings highlight the need for broader strategies targeting multiple nodes within the IGF signaling pathway.^80-82^

Despite promising preclinical findings, the clinical efficacy of IGF-1R-targeted monoclonal antibodies has generally been modest in unselected patient populations. This limited therapeutic benefit is largely attributed to the lack of robust predictive biomarkers, compensatory activation of insulin receptor isoform A (InsR-A), signaling crosstalk with other receptor tyrosine kinases, and tumor heterogeneity.^79-81^ These observations underscore the importance of biomarker-guided patient stratification and molecularly selected therapeutic strategies to enhance the clinical efficacy of IGF-1R-targeted therapies. Sustained cytokine signaling connects chronic inflammation to IR and further promotes tumor progression. Targeting pro-inflammatory cytokines with mAbs represents an additional therapeutic approach. Inhibiting IL-6 or IL-6R may reduce JAK–STAT3-driven proliferation, angiogenesis, and immunosuppression. TNF-*α* inhibition may suppress NF-κB and JNK signaling and improve insulin sensitivity. Monoclonal antibodies against IL-1β may inhibit cytokine amplification and inflammasome-driven inflammation within the TME. Overall, cytokine-targeted antibodies may help reduce the inflammatory microenvironment that supports dysregulated metabolism and tumor growth.^49^ ^,^ ^50^ ^,^ ^83^

Immune evasion is driven by metabolic changes and cytokine activity in the TME. Therapeutic mAbs against immune checkpoints (e.g., PD1, PDL1, CTLA4) have been a game changer in oncology by restoring anti-tumor immunity. In the context of IR, the combination of elevated cytokine levels and metabolic stress leads to dysfunctional T cells and further expansion of immunosuppressive cell types. Immune checkpoint inhibitors have the opposite effect by stimulating cytotoxic T lymphocytes and boosting immune-mediated tumor killing. Furthermore, the TME can rapidly change tumor metabolism and can also influence the response to checkpoint blockade, creating the need for combination therapies.^84-86^ Although monoclonal antibody therapies are highly specific, there are many mechanisms of resistance. One concern is pathway redundancy, in which inhibition of one signaling pathway leads to compensatory activation of others. For instance, inhibition of IGF-1R could increase signaling through InsR-A or other growth factor receptors.^87^ Moreover, the persistent activation of downstream tumor signaling molecules, including STAT3 and components of the PI3K-Akt-mTOR pathway, could sustain tumor viability and a lack of receptor signaling. Resistance mechanisms in the TME, such as those mediated by cytokines and by metabolic and immunologically compromised alterations, may also contribute to this resistance. The described mechanisms of resistance strongly emphasize the need to develop multiple combination-targeted therapies that act at both the upstream and downstream parts of oncogenic signaling pathways.^88-92^ Integrating approaches to improve therapeutic effectiveness is an area of research to facilitate overcoming resistance. For instance, mAbs may be used in tandem with metabolic inhibitors that affect PI3K–Akt–mTOR signaling, JAK–STAT inhibitors, or modulators of glucose and lipid metabolism. Also, the combination with immune checkpoint inhibitors has an additional effect of restoring immune function and reducing tumor growth. Another area of research is improving the targeting and bioavailability of monoclonal antibodies through an innovative nanotechnology-based delivery system. Potential predictive biomarkers, such as circulating cytokine levels or receptor expression profiles, will support the personalization of therapeutic approaches. mAbs targeting the IR–tumor axis is an innovative area of research in cancer therapy. However, translating this will require a greater understanding of metabolic signaling, the immune system, and tumor interactions. The key monoclonal antibody targets, their mechanisms of action, and associated signaling pathways are summarized in Table 2.

**Table 2.** Targets for monoclonal antibodies in the IR–tumor axis.

Target	Description	References
IGF-1R	A receptor tyrosine kinase that mediates IGF-driven cell proliferation and survival via the PI3K–Akt–mTOR and the MAPK pathways. mAbs offer high specificity and inhibit tumor growth by blocking ligands and downstream signaling. However, therapeutic efficacy is frequently compromised by downstream compensatory activation of InsR-A and the emergence of resistance.	[93,94]
IL-6/IL-6R	A major cytokine signaling pathway, the JAK–STAT3 pathway, promotes tumor growth, blood vessel formation, and immune evasion. This pathway has been targeted with some success, as it reduces STAT3 activity and slows tumor growth. However, due to the redundancy in cytokine signaling, therapeutic options may be limited.	[49]
TNF-α	A pro-inflammatory cytokine that activates NF-κB and JNK signaling, promoting IR and driving tumor progression. Blocking this cytokine improves chronic inflammation and metabolic signaling, although the potential for systemic immunosuppression and an increased risk of infection remain concerns.	[45]
IL-1β	IL-1β is an inflammasome-associated cytokine that activates the NF-κB and MAPK pathways and enhances the inflammatory response in the TME. IL-1β inhibition attenuates cytokine amplification and the inflammatory response, but, due to compensatory pathways, the direct anti-tumor effects are minimal.	[52]
PD-1/PD-L1	An immune checkpoint pathway that inhibits T-cell activation and promotes immune evasion by tumors. Monoclonal antibodies can reinstate anti-tumor immunity; some patients experience lasting responses. Efficacy is highly variable, and immune-related adverse events can occur.	[84]
CTLA-4	An immune checkpoint receptor involved in regulating early T-cell activation and immune tolerance. The inhibition of this receptor improves T-cell priming, and when combined with other immunotherapies, a pronounced effect is observed. However, as monotherapy, it is associated with increased toxicity and limited therapeutic activity.	[84]
VEGF	An essential factor in regulating angiogenesis, facilitating the supply of blood vessels and nutrients to tumors. Blocking VEGF reduces angiogenesis and tumor expansion. However, resistance may arise from other adaptive angiogenic mechanisms, and the formation of collateral blood vessels remains a common complication.	[85]

## 5. Resistance mechanisms and combination strategies

IR, chronic inflammation, and TME remodeling have been identified as intricate networks of signaling axes involved in cancer progression. Within this context, mAbs have become highly selective therapeutic agents targeting extracellular ligands, receptors, and immune checkpoints involved in the IR–tumor axis.^23^ mAbs, in contrast to standard therapies, can home in on and disrupt key signaling nodes, thereby interrupting the interplay between metabolic dysregulation and cancer-associated inflammation. Table 3 summarizes monoclonal antibody targets, their therapeutic roles, and their pros and cons within the IR–tumor axis. Targeting IGF-1R and other components, such as cytokine signaling (IL-6, TNF-*α*, IL-1β) and immune checkpoint and angiogenesis pathways, can arrest some of the signaling pathways that promote tumor growth.^80-84^ However, the therapeutic effect is often attenuated by compensatory pathway activation, metabolic flexibility, and resistance in the TME. This table reinforces the importance of multifaceted and combinatorial approaches to achieve a sustained anti-tumor effect. The IGF-1R, a key mediator of the mitogenic effects of hyperinsulinemia, constitutes a major point of interest within this axis. mAbs directed against IGF-1R, namely figitumumab and ganitumab, are designed to prevent ligand binding and subsequent activation of the PI3K–Akt–mTOR and MAPK pathways. They are designed to diminish tumor cell growth, survival, and metabolism. The potential clinical benefit remains to be fully realized, primarily due to compensatory signaling and the activation of InsR-A, which is inhibited by IGF-1R inhibition. The persistence of downstream pathways, particularly STAT3 and Akt, contributes to resistance, exemplifying the limitations of a singular focus.^95^ ^,^ ^96^

**Table 3.** Monoclonal antibodies in the IR–tumor axis: roles, limitations, and resistance.

Monoclonal antibody	Target	Description	References
Figitumumab	IGF-1R	Mechanistically, this strategy is relevant to the IR–IGF axis, as it attempts to inhibit IGF-1R–dependent proliferative signaling, which is associated with hyperinsulinemia and abnormal IGF activity in cancer. Targeting IGF-1R has a clear and strong rationale based on preclinical concepts and data. However, due to insufficient clinical efficacy and the potential risks of metabolic toxicity, such as hyperglycemia, IGF-1R-targeted therapies remain outside of standard clinical practice in oncology. Moreover, therapeutic resistance often arises from InsR-A, the PI3K–Akt pathway, and receptor interactions within the EGFR/HER family, underscoring the redundancy of oncogenic signaling pathways.	[93,97]
Ganitumab	IGF-1R	Focused on solid malignancies with selective blocking of IGF-1R signaling, which is applicable for IGF-dependent cancers; however, it is not a recognized routine cancer treatment. Due to the difficulty of biomarker determination, the clinical effectiveness is inconsistent, with resistance due to insulin InsR compensation, an escape in the downstream pathway (e.g., PI3K–Akt), and multidimensional IGF dependence.	[97,98]
Cixutumumab	IGF-1R	Trialed a monoclonal antibody targeting IGF-1R to block a critical growth and survival receptor involved in IR-related tumor growth. The clinical use of Cixutumumab is limited due to variable antitumor activity. The lack of durability of response to Cixutumumab as monotherapy is mainly due to compensatory activation of InsR-A, in addition to sustained downstream activation of STAT3 and MAPK signaling, resulting in adaptive signaling reprogramming and development of resistance.	[98]
Tocilizumab	IL-6R	A clinically approved anti-IL-6R monoclonal antibody is particularly relevant in malignancies characterized by IL-6–JAK–STAT3 pathway dependence, which mitigates inflammation-driven tumor progression, angiogenesis, and the associated metabolic reprogramming with IR. Even with its mechanism of action, Tocilizumab is limited in its therapeutic applications due to the presence of cytokine redundancy, persistent NF-κB activation, compensatory inflammatory pathway activation, and the immune suppression associated with increased risk of infection.	[99]
Canakinumab	IL-1β	An anti-IL-1β antibody relevant to inflammasome-driven metabolic inflammation has been approved; it blocks NLRP3–IL-1β signaling and thus decreases cytokine cascade amplification and protumor inflammatory signaling in the TME. As a monotherapy, it lacks significant direct tumoricidal activity, is potentially very expensive, has a high risk of infection, and resistance is mediated by compensatory IL-6/TNF-*α* pathways, persistent inflammasome-independent inflammation, and downstream signaling escape mechanisms.	[100]
Bevacizumab	VEGF-A	An FDA-approved antibody targeting multiple solid tumors that binds to VEGF-A to inhibit angiogenesis, decrease vascularization of tumors, and, in some cases, enhance the delivery and therapeutic effect of companion-administered therapies. They are linked with hypertension, thrombosis, bleeding, proteinuria, and impaired wound healing. Resistance develops through other pro-angiogenic signals, vessel co-option, hypoxia-driven adaptive mechanisms, and stroma remodeling.	[101]
Nivolumab	PD-1	An immune checkpoint inhibitor with FDA approval, this drug treats several types of cancer. It blocks PD-1 and reactivates T-cell anti-tumor activity, resulting in durable immune responses in patients who respond. Associated with immune-related adverse events and variable efficacy depending on tumor type and microenvironment; resistance arises due to persistent T-cell exhaustion, impaired antigen presentation, immunosuppressive TME, and metabolic competition within the TME.	[102]
Pembrolizumab	PD-1	An FDA-approved checkpoint inhibitor used in numerous malignancies that locks PD-1 and boosts T-cell anti-tumor activity, leading to sustained clinical responses in certain patient subsets. Various immune-related adverse events may occur, and it is not effective in all tumors that are metabolically inflamed. PD-1 resistance mechanisms include PD-L1–independent escape, T-cell activity depletion, and the emergence of alternative checkpoints.	[103]
Atezolizumab	PD-L1	A PD-L1 antibody for PD-L1-mediated immune suppression approved by the FDA as a therapeutic for a limited number of cancers. Restores anti-tumor immunity. When used in combination with other therapeutics, it can increase efficacy and the clinical immune response. Clinical immune response can be unpredictable and may involve immune-related side effects. Among the many suggested mechanisms of resistance are immune-related toxicities and clinical responses, including cytokine-mediated immune escape, activation of other immune checkpoints, and resistant stromal niches within the TME.	[104]
Avelumab	PD-L1	An FDA-approved PD-L1 antibody for specific types of cancer that reverts anti-tumor immunity by blocking PD-L1 signaling and, in certain types of tumors, provides benefit as a maintenance therapy. Immune-related adverse toxicities are possible. Resistance remains due to comparable checkpoint escape mechanisms and due to a sustained, metabolically hostile TME	[105]
Ipilimumab	CTLA-4	An FDA-approved checkpoint inhibitor targeting CTLA-4; improves initial stimulation and activation of T-cells and provides additional benefits when used in combination with PD-1/PD-L1 blockade. Compared to monotherapy with PD-1/PD-L1 blockade, it is associated with a greater immune-related adverse event burden; as a single agent, it demonstrates limited activity in numerous malignancies, which can be attributed to resistance from a combination of alternative inhibitory pathways, a suppressive microenvironment of the tumor, and adaptive immune escape.	[106]

IR tumors with chronic inflammation also benefit from mAbs targeting cytokines. IL-6 is a key driver of tumor growth and immune evasion and a major player in the JAK-STAT3 pathway. IL-6 antagonists and IL-6 receptor antagonists, such as tocilizumab and siltuximab, inhibit STAT3 and reduce inflammatory signaling. These treatments are particularly useful in the presence of elevated IL-6 in the TME; however, the presence of TNF-*α* and IL-1β signaling in parallel pathways often causes limitations due to cytokine redundancy.^107-109^ Likewise, TNF-*α* inhibitors, including infliximab and adalimumab, block the NF-κB and JNK pathways and lessen the signaling associated with chronic inflammation. These agents are useful in managing systemic inflammation, but because tumor evolution is a complex phenomenon, their direct anti-tumor effects are, at best, minimal. In addition, the detrimental effects of systemic immunosuppression and increased risk of infections are considerable. An additional means of managing the inflammatory component of the TME is to target the inflammasome activator IL-1β. Canakinumab, a monoclonal antibody, blocks IL-1β signaling, thereby attenuating NF-κB and MAPK activation and reducing the subsequent cytokine cascade. While this approach is excellent at controlling inflammation, its direct effect on tumor regression remains very limited because tumor cells can use alternative cytokine pathways to promote their growth and survival.^110-112^ While this approach may significantly reduce inflammation, the direct anti-tumor effect is often limited because tumor cells can exploit other pro-cytokine networks to continue proliferating and surviving.^113^ ^,^ ^114^ Unlike strategies targeting cytokines, immune checkpoint inhibitors have shown considerable clinical efficacy in treating cancer. mAbs against PD-1, PD-L1, and CTLA-4 restore T-cell activity and boost anti-tumor immune responses. In insulin-resistant tumors, metabolic competition and chronic inflammation that inhibit immune responses can be partially mitigated by checkpoint blockade, partially restoring the immune response. However, therapeutic responses are variable. Metabolic restrictions in the TME, such as T-cell functional impairment due to glucose scarcity and lactate overload, continue to limit T-cell function despite checkpoint inhibition. In addition, immune-related adverse events are a significant hindrance to clinical use.^95^ ^,^ ^96^ ^,^ ^107^ Angiogenesis-targeting monoclonal antibodies, especially those that bind to vascular endothelial growth factor (VEGF), are important in oncological treatments. Bevacizumab, for instance, hinders tumor vascularization and, ultimately, tumor growth by restricting nutrient supply. Although this is beneficial in some circumstances, resistance often arises via other angiogenic pathways and adaptive responses to hypoxia. These constraints illustrate how difficult it is to target the TME, as numerous compensatory mechanisms operate concurrently.^115-117^

The development of resistance poses a significant challenge for monoclonal antibody therapy. Tumor cells are highly adaptive, activating distinct signaling pathways to evade lethal responses. For instance, if IGF-1R is inhibited, there may be greater dependence on insulin receptor signaling, or other pathways may become active, including EGFR or HER2. Targeting a cytokine may be circumvented by upregulation of another inflammatory mediator. Immune checkpoint resistance may also result from insufficient antigen presentation or the presence of a persistent immunosuppressive cell population.^118-123^

The development of new therapeutic combinations is an active area of research aimed at addressing the above challenges. The combination of monoclonal antibodies with metabolic blockers, such as PI3K–Akt–mTOR or JAK–STAT inhibitors, may enable targeting of multiple targets along the IR–tumor axis. Moreover, combining immune checkpoint inhibitors with therapies targeting cytokines may augment anti-tumor immunity by modulating both immune and metabolic pathways. Other technologies, such as biomarker-guided therapy and nanotechnology for modified delivery systems, further improve the accuracy and effectiveness of monoclonal antibody treatments. Overall, monoclonal antibody therapeutics offer a unique opportunity to target the IR–tumor axis, but their successful implementation requires addressing the complex systems and redundancies within the TME.

## 6. Conclusion

IR involves a disruption of systemic and cellular energy balance, along with changes in inflammatory and growth-factor signaling. Selective signaling bias, in which the metabolic insulin signaling pathways are turned off, while the mitogenic and survival signaling pathways driven by insulin-IGF signaling remain preferentially activated, is a central phenomenon. These conditions produce a chronic excess of nutrients and signaling, which together foster an environment that increases the risk of cancer development, characterized by intratumoral paradoxical lipid accumulation, immune cell infiltration, activated immune cells and cytokines, resistance to cell death, and changes in cell type and function. These facets interact and foster IR, tumor growth, immune evasion, and metabolic dysfunction. This complexity highlights the importance of considering interconnected metabolic, inflammatory, and growth-factor signaling pathways when developing therapeutic strategies for insulin resistance-associated cancers. Emerging evidence suggests that combining metabolic interventions with targeted therapies and biomarker-guided patient stratification may improve therapeutic outcomes. However, the clinical effectiveness of these approaches, including combination strategies and individualized treatment based on immunometabolic biomarkers, requires further prospective clinical validation before routine implementation in precision oncology.

## Data Availability

No new data were generated or analyzed in this study. Data sharing is not applicable to this article.
